# Flourishing Together: The Longitudinal Effect of Goal Coordination on Goal Progress and Life Satisfaction in Romantic Relationships

**DOI:** 10.1007/s41042-023-00089-3

**Published:** 2023-03-10

**Authors:** Orsolya Rosta-Filep, Csilla Lakatos, Barna Konkolÿ Thege, Viola Sallay, Tamás Martos

**Affiliations:** 1 Doctoral School, Semmelweis University, Budapest, Hungary; 2 Faculty of Health Sciences, University of Miskolc, Miskolc, Hungary; 3 Waypoint Research Institute, Waypoint Centre for Mental Health Care, Penetanguishene, L9M 1G3 Canada; 4 Department of Psychiatry, University of Toronto, Toronto, Canada; 5 Institute of Psychology, University of Szeged, Szeged, Hungary

**Keywords:** Romantic relationships, Personal projects, Life satisfaction, Goal coordination

## Abstract

Goal pursuit shapes people’ everyday experiences and is deeply embedded within close relationships. Several studies have shown that goal support from romantic partners facilitates goal progress, and individual goal progress contributes to wellbeing. However, few pieces of research have examined the whole process, how efficient goal coordination in a romantic relationship contributes to life satisfaction through goal progress. In these studies, short time frames were used and only one aspect of goal coordination was examined. To generate more complex, long-term understanding we collected data from 148 married or cohabitating Hungarian heterosexual couples (mean age 39.71 ± 10.40 and 38.57 ± 10.00 years for men and women, respectively) in a two-wave longitudinal study with a year-long time window. Both partners individually completed an adapted version of the Personal Project Assessment and evaluated four chosen projects associated with project coordination (emotional support, communication, and cooperation) at baseline, and project attainment (progress, success, satisfaction) in the follow up. Life satisfaction was assessed during both waves. Results from the actor–partner interdependence mediation modeling revealed complete mediation, where project coordination increased project attainment one year later, and consequently associated with higher life satisfaction for both partners. The direct effect between project coordination and life satisfaction remained nonsignificant. This association indicates that for long-term life satisfaction, it is crucial to experience better goal outcomes as the result of the couple’s collaborative effort.

## Introduction

Goal pursuit shapes how people experience their lives. They may strive to be promoted, organize a vacation, or lose some weight, while also trying to be good partners to their loved ones. Progressing with or attaining personal goals increases individual well-being (Brunstein, 1993; Kaftan & Freund, 2018; Klug & Maier, 2015; Sheldon & Kasser, 1998). Although a vast literature exists about the individual aspects of goal progress (see for review Diefendorff & Lord, 2008; Milyavskaya & Werner, 2018), goal pursuit never happens in isolation as it is embedded in a socioecological context of personal relationships (Fitzsimons & VanDellen, 2015; Little, 2015). There is growing focus on what the support of close others, especially romantic partners, adds to the pursuit of individual goals (see for meta-analysis Vowels & Carnelley, 2022), but only a few studies have linked goal progress to individual wellbeing in the context of romantic relationships, focusing on the effect of support under a shorter timeframe (Jakubiak & Feeney, 2016; Koestner et al., 2012). To obtain a more complex understanding of how romantic partners facilitate each other’s goal progress, and consequently wellbeing, we studied the personal goals of couples living together in a committed relationship. Below, we detail the theoretical context concerning how the relationship context affects goal strivings. We focus on how a romantic partner can facilitate individual goal progress and wellbeing.

## Goal Pursuit in Relationships

Goals can be defined as “a cognitive representation of a desired end state that a person is committed to attain” (Milyavskaya & Werner, 2018, p. 163). Several approaches exist concerning of similar, but conceptually different types of goal constructs (Little, 1989). One of the most common approaches are personal projects, defined as “a set of interrelated acts extending over time, which is intended to maintain or attain a state of affairs foreseen by the individual” (Little, 1983, p. 276). Although there is a nuanced difference, the terms are often used interchangeably in literature, and the different methods result in mostly similar responses (Milyavskaya et al., 2022). Thus, in the following we use the two terms interchangeably, preferring goals to describe general concepts and associations, and projects when it is methodologically more relevant.

One of the most comprehensive theories about the interpersonal framework of goal striving, Transactive Goal Dynamics Theory (TGD Theory), conceptualizes people in close relationships as one self-regulating unit based on the strong interdependence of members’ goal setting, pursuit, and outcomes (Finkel & Fitzsimons, 2019; Fitzsimons & Finkel, 2018; Fitzsimons et al., 2015). Thus, goal striving can be better understood through interdependence and interaction in association with individuals’ close relationships, from which romantic relationships are primarily relevant. Interdependent partners attain their individual goals better (in other words, they experience transactive gain) when they manage to align their goal-relevant resources, which process is called goal coordination (Fitzsimons & Finkel, 2018).

Successful goal attainment in turn is part of a virtuous cycle, as members feel closer and are more satisfied in a relationship where the partner is supportive (Brunstein et al., 1996; Fitzsimons & Fishbach, 2010; Jakubiak & Feeney, 2016; Kaplan & Maddux, 2002).

Although the focus of TGD Theory are the interpersonal aspects of goal-striving processes and relationship satisfaction, it addresses the role of individual wellbeing (Fitzsimons et al., 2015). The authors propose that when people experience that their goals are progressing well, they feel and behave more positively, are more instrumental to each other’s goals, which helps them to be better partners (Fitzsimons et al., 2015). Supporting this assumption, Moore and Diener (2019) found that individuals with higher subjective wellbeing rated their partners as more helpful and were more satisfied with their relationship, and were associated with partners who themselves reported higher relationship satisfaction. In a meta-analysis Proulx et al. (2007) also found a close connection between marital quality and personal wellbeing, suggesting the opposite causal direction – better marital relationships may increase personal wellbeing.

## Behavioral Aspects of Goal Coordination

Goal coordination is efficient when the goal pursuit of one spouse facilitates the other spouse’s goal pursuit; the pursuit of one goal serves multiple goals; and the couple leverage the strengths and preferences of the partnership (Fitzsimons et al., 2015). TGD theory details the prerequisite traits and skills for efficient goal coordination but is ambiguous about the everyday behavioral mechanisms through which these traits and skills are transformed into well-coordinated goals. In the following, we propose three such mechanisms through which efficient goal coordination can emerge: namely, support, communication, and cooperation, all attached to members’ individual goals.

## Goal Support

The effect of support on goal progress and wellbeing in the context of a romantic relationship is well established in the literature. Effective support should be responsive to the recipient’s needs (Finkel & Fitzsimons, 2019; Fitzsimons et al., 2015; Zee et al., 2020), respect the recipient’s autonomy, feelings and perspective (Koestner et al., 2012), and provide emotional comfort and facilitate resolution (Feeney & Collins, 2015). In general, different theoretical approaches emphasize emotional support as an integral element of any effective support. According to a recent meta-analysis, emotion-focused support (affirmation and responsiveness) has the greatest effect on goal progress compared to practical and negative support (Vowels & Carnelley, 2022). Thus, in the following we focus on the effect of emotional support in longitudinal studies with romantic couples.

Emotional support has been found to facilitate progress on relationship goals (Sadikaj et al., 2015), self-improvement (Overall et al., 2010), and managing the difficulties associated with the COVID-19 pandemic (Vowels et al., 2021). People who receive emotional support pursue more challenging goals and report to greater learning and growth (Feeney et al., 2017). Goal support, in turn, facilitates individual wellbeing for romantic couples (Molden et al., 2009; Soulsby & Bennett, 2015).

Two longitudinal studies connected both emotional support and goal progress to individual wellbeing in romantic relationships. In a three-month longitudinal study, autonomy support (acknowledgement of the recipient’s feelings and perspectives through the encouragement of their choices and options) was identified as positively related to goal progress over time, but autonomy support did not predict change in subjective wellbeing, even though significant associations were found in the other studies reported in the article with non-romantic dyads (Koestner et al., 2012). A one-week diary study investigated the mediating effect of goal progress between emotional support and individual wellbeing (Jakubiak & Feeney, 2016). Secure base support (encouragement of exploration and providing help only if needed) and wellbeing were linked directly and indirectly through daily goal progress. Secure base support predicted goal progress on the same and even the following day. In turn, daily goal progress increased subjective wellbeing on the same and the following day as well, while both associations were stronger for wives. The gender differences were unexpected and contrast with the findings of previous studies on emotional support and goal progress, where no gender differences emerged (Overall et al., 2010; Sadikaj et al., 2015).

## Communication

To be capable of giving proper emotional support, spouses need the opportunity to learn about their partners’ goals, preferences, and skills, which is facilitated through communication about goals (Fitzsimons et al., 2015). However, the literature about the frequency of goal communication and its relation to subjective wellbeing is scarce. In general, positive communication is a reliable predictor of couples’ relationship quality (see for a meta-analysis Kanter et al., 2021). Sharing positive events to significant others raises positive affect and satisfaction with life. When such sharing is responded to by an actively and constructively responsive romantic partner, it raises relationship quality as well (Gable et al., 2004). Moreover, the quantity of communication contributes to relationship satisfaction, even after controlling for its positive quality (Emmers-Sommer, 2004).

## Cooperation

Besides emotional support and communication, the third mechanism through which partners can align and integrate their efforts and resources is through cooperation in activities which facilitate the partner’s goal progress. Cooperation can be an integral part of communication, and include an active, constructive attitude to discussion or problem solving. Cooperating negotiation strategies facilitate couples’ emotion regulation. Romantic partners’ emotions synchronize when they express high levels of cooperation while discussing topics relevant to romantic relationships (Randall et al., 2013). Elderly coupes used more collaborative problem-solving strategies in their joint goals (discussing the obstacle or dealing with the problem together), which they perceived as highly effective. Progress on joint goals in turn decreased negative affect (Hoppmann & Gerstorf, 2013). Shared selves of elder couples (which can be considered a goal concept) lead to better wellbeing through better enjoyment of collaboration which was robust for marital quality and subjective health (Schindler et al., 2010).

Joint activity can be considered as an active way a couple cooperates in their goals, which facilitates goal progress as well. In newly dating relationships, perceived goal congruence (spending time together in health-related goal activities) increased goal commitment over time (Marshall & Gere, 2022). A health-related diary study with romantic couples showed that joint activity accounted for almost half of the effects of support on the recipient’s daily activity. The authors argue that joint activity may be an important way through which support helps goal implementation in everyday life (Berli et al., 2018).

## The Present Study

There is a separate but well-established positive connection between support and goal progress (Feeney et al., 2017; Overall et al., 2010; Sadikaj et al., 2015; Vowels & Carnelley, 2022) and between emotional support and individual wellbeing (Molden et al., 2009; Soulsby & Bennett, 2015) in romantic relationships. However, research has attempted to connect all three aspects and examine how emotional support contributes to wellbeing through goal progress using a limited time window of a week (Jakubiak & Feeney, 2016) and three months (Koestner et al., 2012). While general tendencies emerged as studies universally emphasize the importance of emotional support, the influence of other aspects of goal coordination remain ambiguous. Several studies reported no gender differences (Overall et al., 2010; Sadikaj et al., 2015), while Jakubiak and Feeney (2016) reported stronger effects for women.

In the present study we aim to go beyond the established associations of emotional support, goal progress, and wellbeing (Jakubiak & Feeney, 2016; Koestner et al., 2012; Vowels & Carnelley, 2022), and to integrate emotional support with other aspects of goal coordination – namely, partners’ communication about personal goals (Emmers-Sommer, 2004; Gable et al., 2004) and their cooperation in relation to such goals (Berli et al., 2018; Hoppmann & Gerstorf, 2013; Marshall & Gere, 2022; Schindler et al., 2010). To this end, we used Personal Projects Analysis, which provides a personally meaningful measurement of goals connected to behavior in an everyday life context (Little, 2015). Although we assess the individually pursued personal projects of both members of a couple, these projects reflect on the relationship context in which the goal pursuit happens, as demonstrated in previous studies (Martos et al., 2019a, b). We extend previous research by using a one-year longitudinal study that enables us to go beyond daily dyadic adjustments and to examine the effects of goal coordination and progress over a wider time frame.

**Fig. 1:**
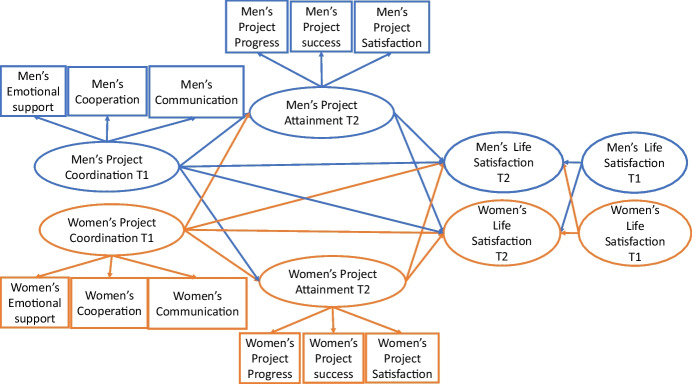
Proposed model defining and linking goal coordination, goal progress and life satisfaction. T1: first wave; T2: second wave. The covariances between measurement errors of paired items are omitted for clarity.

Based on the literature, we expect goal coordination to facilitate goal attainment and, in turn, life satisfaction. By studying both members of a romantic couple, we can test potential partner effects too, where we expect individual goal progress to have a spillover effect on partners’ life satisfaction. Figure 1 depicts the proposed theoretical model of goal coordination to goal progress and life satisfaction for both partners.

## Methods

### Participants and Procedure

Experienced research assistants from a survey firm recruited 215 heterosexual couples from Hungary. The eligibility criteria were that couples should: 1) have been cohabitating for at least one year, 2) be between 25 and 65 years old, 3) have no previous history of psychiatric disorders in the past five years, and that 4) at least one spouse has active working status. Two couples were excluded from the analysis for not meeting at least one of the criteria. For our final sample, 148 couples were available for the second wave of the study, while 65 couples refused or were unavailable.

The mean age was 39.72 years for men (SD = 10.40) and 38.57 years for women (SD = 10.00). The average relationship length was 17.10 years (SD = 9.99 years). Regarding relationship status, 64 couples (43.24%) were cohabiting without marriage, and 84 were married (56.75%). More than two-thirds (70.27%) of men and 68.92% of women reported to having at least one biological child (m = 1.41 for men and m = 1.42 for women, respectively). 18.07% of participants had earned college diplomas (16.89% of men and 19.26% of women), 63.85% had earned a high-school diploma (66.22% of men and 61.49% of women) and 16.89% had completed only primary education (20.95% of men and 12.84% of women).

Prior to the data assessment we acquired ethical approval from Semmelweis IRB (SE TUKEB). Potential participants were contacted at their homes. After both partners provided their informed consent, which included their inclusion in a one-year follow up, the interviewer administered the questionnaire pack and explicitly instructed the spouses to complete the assessment procedure separately. Then the interviewer left participants to fill out the survey by themselves, and recollected completed questionnaire pack at a later date agreed by phone. All data collected were handled confidentially. All partners that participated in the study voluntarily and received a book voucher for their contribution (6000 HUF, ~ US$20 per couple) in each wave of the study. Data sampling occurred from 2013 to 2014, which makes the sample unaffected by COVID-19 pandemic.

The same procedure was repeated one year later. To examine potential drop-out bias, we compared the sample of retained to lost couples. There were no differences in the goal-related or well-being variables under analysis at baseline. Regarding the demographic variables, we detected no difference in age, relationship status, numbers of children, or level of education, but couples who withdrew from the study had relationships shorter by three years on average (*t*(182) = -2.236, p = 0.027).

### Measures

#### Demographic Variables

The demographic information section of the survey included questions on age, gender, relationship length and status, number of children and education.

#### Personal Project Assessment

We assessed goal-related personal and relationship experiences using an adapted version of Little's (1983) Personal Project Assessment (PPA) procedure. At the first wave (Time 1), we asked participants to generate a list of their personally important projects, defined as: ‘the goals and strivings that you are currently working on in your everyday life.’ In the next step, they were asked to select the four most relevant projects from the list. Examples of projects included ‘renovating our house’ or ‘going on vacation.’ Finally, participants were asked to rate each of the four projects according to a series of predefined criteria on a seven-point Likert scale, concerning their project-related individual and relational needs and experiences. The relevant aspects are detailed in the following two sections. The four project ratings were aggregated into a composite project score for each aspect under investigation and entry into the subsequent analysis.

##### Project Coordination

Participants evaluated how well they and their partner coordinate their efforts and resources regarding each of their selected four projects. Project coordination was assessed at the first wave of the study (Time 1), using three items – based upon communication (‘How frequently do you communicate with your partner about this project?’), cooperation (‘How frequently do you cooperate with your partner on this project?’) and the partner’s perceived emotional support (‘My partner supports me emotionally [e.g., accepting, caring] in this project.’). The endpoints of the Likert scales were noted as 1 = ‘very rarely’ and 7 = ‘very often’. The internal consistency of this scale was acceptable (α = 0.754 for men and 0.781 for women partners).

##### Project Attainment

During the second wave (Time 2), participants were presented the four projects they selected at the first wave and they were instructed to evaluate, how much they attained from each project using the following items: ‘How far have you progressed with this project?,’ How successful were you in the accomplishment of this project?’ (1 = Very little; 7 = Very much) and ‘Taken together, how satisfied are you with the way things have gone with this project over the past year?’ (1 = Not satisfied at all; 7 = Completely satisfied). The internal consistency of this scale was excellent for both genders (α = 0.972 for men and 0.965 for women partners).

#### Satisfaction With Life Scale

The Satisfaction With Life Scale (SWLS; Diener et al., 1985; Hungarian adaptation: Martos et al., 2014) is a five-item measure for assessing overall general satisfaction with life, where respondents indicate their degree of agreement on a seven-point Likert scale (1 = strongly disagree, 7 = strongly agree). One sample item is ‘In most ways my life is close to my ideal.’ SWLS was assessed in both waves. The alpha coefficient indicated the good-to-excellent reliability of this scale for both genders in both waves (α = 0.910 for men and 0.904 for women partners at Time 1 and α = 0.899 for men and 0.909 for women partners at Time 2). The two assessments showed moderate correlation for men (r = 0.461, *p* < 0.001) and medium correlation for women partners (r = 0.565, *p* < 0.001).

#### Overview of the Analytical Process

To ensure measurement invariance across genders and timepoints, as suggested by Gareau et al. (2016), two separate series of confirmatory factor analyses (CFA) were conducted on personal project and life satisfaction variables. We utilized chi-square difference test for comparing the models, but since this approach may lead to biased decisions based on the sensitivity of Chi-square statistics (χ^2^) (Yuan & Chan, 2016), we extended our process with Chen's (2007) recommendation concerning smaller samples with unequal groups and considered those models invariant that had a change of Comparative Fit Index (CFI) less than 0.005 and change of Root Mean Square Error of Approximation (RMSEA) not larger than 0.010.

The non-independence of dyadic data was accounted for by applying an actor-partner interdependence mediation model (APIMeM; Ledermann et al. (2011)), which is an extended version of the actor-partner interdependence model (APIM; Cook & Kenny, 2005). In the basic APIM, the effect of a person’s predictor variable on their own (actor effect) and their partner’s (partner effect) outcome variable are estimated in one model simultaneously, while controlling for the covariance and the correlations between the same variables from both partners. Project coordination from both partners were entered as predictors in the model, the pairs of life satisfaction were regarded as outcomes. Project attainments were added as mediators. We used life satisfaction on T1 as a control for life satisfaction in T2. Following the recommendations of Kenny and Ledermann (2010), first, we estimated the saturated distinguishable model. We imposed constraints on all direct effects in the next step to test for a gender effect. Then we examined the indirect effects of project attainment on the associations between project coordination and satisfaction with life (Ledermann et al., 2011).

Because of the high complexity of the model, a sensitivity analysis was conducted for the final APIMeM, where we used the sum scores instead of latent factors for SWLS’, based on its high internal consistency (see Appendix 13.).

Structural equation modeling (SEM) was used for all analyses. We evaluated model fit for baseline models and hypothesis testing based on CFI, RMSEA, and the Standardized Root-Mean-square Residual (SRMR). The model was considered to fit the data well with a CFI value of 0.90 or higher, an RMSEA value of 0.06 or lower, and a SRMR value of 0.08 or lower (Kline, 2010). All analyses were conducted using MPlus (version 7.11; Muthén & Muthén, 2012) and we implemented full-information maximum likelihood (FIML) estimation with 5,000 bootstrapping samples to control for the non-normality in the data and for testing conditional indirect effects.

## Results

### Preliminary Analyses

Descriptive statistics and correlations of the Personal Project Assessment variables and SWLS are shown in Table 1. As expected, items related to project coordination were significantly correlated (r = 0.397 to 0.699 for men and 0.431 to 0.712 for women, respectively; *p* < 0.001), while project attainment items were positively related as well (r = 0.902 to 0.939 for men and 0.889 to 0.917 for women partners, respectively; *p* < 0.001). Following the hypothesized pattern, items related to project attainment showed significant positive correlation with SWLS at the second wave (r = 0.390 to 0.427 for men and 0.389 to 0.454 for women, respectively; *p* < 0.001). Women’s SWLS was significantly associated with support (r = 0.249; *p* < 0.001) and items related to project attainment (r = 0.211 to 0.251; p = 0.002 to 0.011). Correlation between items of project coordination and project attainment was non-significant in most cases with the exception of men’s communication and success (r = 0.186; p = 0.025). In general, couples had similar expressions in project-related experiences (r = 0.309 to 0.533; *p* < 0.001) and life satisfaction (r = 0.583 for T1 and r = 0.726 for T2; *p* < 0.001) similarly to their partners.

**Table 1:** Correlation matrix and descriptive statistics for the variables

					Men									Women						
					1	2	3	4	5	6	7	8	9	10	11	12	13	14	15	16
Men	Personal Projects T1		Range	SD																
	1	Cooperation	2.25–7	1.125	5.203															
	2	Communication	1–7	1.288	0.699**	4.986														
	3	Support	2.25–7	0.956	0.397**	0.402**	5.774													
	Personal Projects T2																			
	4	Progress	1–7	1.319	0.105	0.145	0.022	4.240												
	5	Success	1–7	1.334	0.136	0.186*	0.074	0.939**	4.195											
	6	Satisfaction	1–7	1.330	0.102	0.146	0.032	0.920**	0.902**	4.204										
	Satisfaction with Life Scale																			
	7	SWLS T1	1–7	1.312	0.058	0.085	0.169*	0.152	0.146	0.156	4.467									
	8	SWLS T2	1–7	1.332	-0.098	-0.096	-0.034	0.401**	0.427**	0.390**	0.477**	4.378								
Women	Personal Projects T1																			
	9	Cooperation	1.5–7	1.094	0.309**	0.249**	0.176*	-0.021	-0.023	-0.006	-0.030	0.063	5.186							
	10	Communication	1.75–7	1.240	0.252**	0.419**	0.141	-0.029	-0.018	-0.001	0.064	0.007	0.712**	4.862						
	11	Support	2.5–7	1.013	0.103	0.135	0.326**	-0.031	-0.008	-0.029	0.156	0.089	0.480**	0.431**	5.716					
	Personal Projects T2																			
	12	Progress	1–7	1.194	0.087	0.036	-0.070	0.495**	0.453**	0.413**	0.125	0.342**	0.048	0.110	-0.064	4.174				
	13	Success	1–6.75	1.210	0.079	0.053	-0.034	0.525**	0.502**	0.442**	0.121	0.322**	0.082	0.130	-0.018	0.917**	4.060			
	14	Satisfaction	1–6.75	1.214	0.109	0.053	-0.093	0.584**	0.548**	0.533**	0.170*	0.348**	0.076	0.132	-0.028	0.889**	0.898**	4.021		
	Satisfaction with Life Scale																			
	15	SWLS T1	1.2–7	1.320	-0.045	-0.061	0.050	0.125	0.141	0.113	0.583**	0.454**	0.053	0.100	0.249**	0.211*	0.251**	0.288**	4.548	
	16	SWLS T2	1–6.8	1.358	-0.014	-0.092	0.060	0.470**	0.479**	0.440**	0.405**	0.726**	0.014	-0.028	0.155	0.389**	0.438**	0.454**	0.583**	4.439

The means are presented diagonally.

For clarity’s sake, aggregated scores for SWL Scales are presented here.

T1 = first wave; T2 = second wave.

*p < 0.05; **p < 0.01.

#### Discriminant Validity and Measurement Invariance

Table 2 presents the comparison of the CFAs with different levels of measurement invariance. The base models confirm discriminant validity (χ^2^ (42) = 38.257, CFI = 1.000, RMSEA < 0.001 for PPA variables and χ^2^ (154) = 258.59, CFI = 0.956, RMSEA = 0.068 for SWLS). The results provide evidence for metric invariance across gender and timepoints for PPA variables (Δχ^2^ (4) = 3.507, p = 0.476, ΔCFI < -0.001, ΔRMSEA < 0.001 for the metric against the configural model; Δχ^2^ (5) = 529.785, *p* < 0.001, ΔCFI = -0.346, ΔRMSEA = 0.263 for the scalar against the metric model) and SWLS (Δχ^2^ (11) = 14.798, p = 0.191 ΔCFI = -0.001, ΔRMSEA = -0.001 for the metric against the configural model; Δχ^2^ (16) = 471.335, *p* < 0.001, ΔCFI = -0.191, ΔRMSEA = 0.078 for the scalar against the metric model).

**Table 2:** Model fit indices for measurement invariance models across time and genders

Models	χ^2^	df	CFI	TLI	RMSEA	SRMSR	Δ χ^2^	Δdf	ΔCFI	ΔRMSEA	ΔSRMSR
Personal Projects											
Step 1. Configural model	38.257	42	1.000	1.004	0.000	0.031					
Step 2. Loading constraint	41.764	46	1.000	1.004	0.000	0.041	3.507	4	0.000	0.000	0.010
Step 3. Scalar model	571.549	51	0.654	0.553	0.263	4.104	529.785	5	-0.346	0.263	4.063
Satisfaction With Life											
Step 1. Configural model	258.59	154	0.956	0.946	0.068	0.051					
Step 2. Loading constraint	273.388	165	0.955	0.948	0.067	0.058	14.798	11	-0.001	-0.001	0.007
Step 3. Scalar model	744.723	181	0.764	0.752	0.145	2.788	471.335	16	-0.191	0.078	2.730

#### Mediating Effect of Project Attainment Between Project Coordination and Life Satisfaction

First, we constrained all direct effects across the dyads to test for gender invariance. We found no evidence for gender effects (Δχ^2^ (8) = 10.43, p = 0.236), and the resulting model had a good fit to the data (χ^2^ (450) = 638.070, CFI = 0.954, TLI = 0.949, RMSEA = 0.053, SRMSR = 0.080), which was retained as final for the interpretation.

**Fig. 2:**
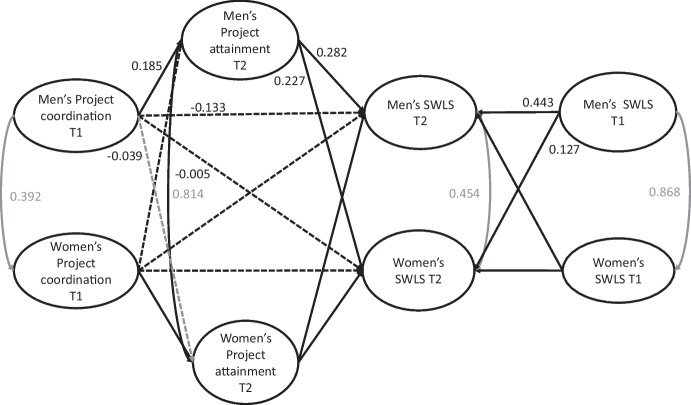
The APIMeM model showing mediated actor and partner effects for life satisfaction. Direct lines: *p* < 0.05; dashed lines: *p* > 0.05. Standardized coefficients are reported. *SWLS* Satisfaction with Life Scale, *T1* First timepoint; *T2*: second timepoint

As Fig. 2 and the upper panel of Table 3 show, the APIMeM analyses revealed positive actor effects for the mediated paths. Higher project coordination resulted in higher project attainment (*b* = 0.185, p = 0.022), which in turn was associated with higher life satisfaction (*b* = 0. 282, *p* < 0.001). The direct path between project coordination and life satisfaction was not significant (*b* = -0.133, p = 0.067). As for partner effects, only the partner’s project attainment was associated with higher life satisfaction (*b* = 0.227, *p* < 0.001). However, it was not significant between the partner’s project coordination and project attainment (*b* = -0.039, p = 0.553) and between the partner’s project coordination and life satisfaction (*b* = -0.005, p = 0.941). All effects for life satisfaction between the first and the second timepoint were significant (*b* = 0.443, *p* < 0.001 for actor and *b* = 0.127, p = 0.042 for partner effects).

**Table 3:** Unstandardized effects for the APIMeM with Project coordination as the independent variable, project attainment as mediator, and life satisfaction as outcome

	b	SE	p	CI (95%)	
				Upper	Lower
Direct effects					
Project Coordination T1—Project attainment T2					
Actor	0.185	0.081	0.022	0.027	0.343
Partner	-0.039	0.066	0.553	-0.17	0.091
Project attainment T2—SWLS T2					
Actor	0.282	0.051	< 0.001	0.183	0.381
Partner	0.227	0.053	< 0.001	0.122	0.331
Project Coordination T1—SWLS T2					
Actor	-0.133	0.073	0.067	-0.275	0.009
Partner	-0.005	0.074	0.941	-0.151	0.14
SWLS T1—SWLS T2					
Actor	0.443	0.071	< 0.001	0.304	0.582
Partner	0.127	0.063	0.042	0.004	0.25
Indirect effects					
Actor					
Total	-0.09	-1.207	0.227	-0.235	0.056
Total Indirect	0.043	1.316	0.188	-0.021	0.107
Actor-Actor	0.052	2.074	0.038	0.003	0.101
Partner-Partner	-0.009	-0.572	0.567	-0.04	0.022
Partner					
Total	0.025	0.331	0.741	-0.124	0.175
Total Indirect	0.031	0.979	0.328	-0.031	0.092
Actor–Partner	0.042	2.052	0.040	0.002	0.082
Partner-Actor	-0.011	-0.583	0.560	-0.048	0.026

Unstandardized coefficients are reported.

*SWLS* Satisfaction With Life Scale.

*T1* First timepoint, *T2* second timepoint.

Indirect effects were tested with bootstrap confidence intervals.

The results from testing the indirect effects supported the two patterns of complete mediation (see the lower panel of Table 3). Both the actor–actor (*b* = 0.052, p = 0.038). and the actor-partner indirect effects (*b* = 0.042, p = 0.040) were significant. All other indirect effects remained nonsignificant.

## Discussion

In the present study, we examined the longitudinal effect of goal coordination on individual life satisfaction through goal attainment of couples who were living together in a committed relationship. By collecting data from both partners at baseline and follow up a year after, we were able to investigate beyond short-term goal adjustments and evaluate prospective associations at the couple level in more detail. The assessment of the relational aspects of personal projects allowed us to capture the unique ways in which the relationship context of pursuing personally meaningful goals contributes to individual life satisfaction.

As expected, a higher level of project coordination resulted in better project attainment one year later. In turn, project attainment also raised life satisfaction for the actors and their partners, even after controlling for baseline life satisfaction. The results support the established link between goal support and goal progress (Feeney et al., 2017; Overall et al., 2010; Sadikaj et al., 2015; Vowels et al., 2021) and between goal progress and subjective well-being (Klug & Maier, 2015). In other words, even though communication, cooperation, and emotional support facilitate only the individual’s goal attainment, attaining one’s goals has benefits on a couple’s level as both partners feel more satisfied with their life as a result. The analysis of indirect effects resulted in complete mediation. This association indicates that for long-term life satisfaction, it is crucial to experience transactive gain and better goal outcomes as the result of the couple’s collaborative effort (Fitzsimons & Finkel, 2018; Fitzsimons et al., 2015). The joint experience surrounding goal coordination is not insufficient, even though goal support was linked to subjective wellbeing in previous cross-sectional studies (Klug & Maier, 2015; Molden et al., 2009; Soulsby & Bennett, 2015).

We found no gender differences in our sample, similar to previous studies which repeatedly found no gender difference in prospective associations between emotional support and goal progress (Overall et al., 2010; Sadikaj et al., 2015). In contrast, Jakubiak and Feeney (2016) found goal support more beneficial for women’s goal progress and wellbeing. Statistical invariance might not be enough evidence for an indistinguishable dyad and should be interpreted cautiously (Fitzpatrick et al., 2016). Further studies with larger sample sizes might be needed to demonstrate a more nuanced process, including gender differences.

## Limitations and Future Directions

While interpteting the results, the following limitations should be noted. First, we implemented a two-wave longitudinal study which cannot grasp the potentially circular relationship between goal coordination and wellbeing. Use of an intensive longitudinal design with more timepoints could more precisely discern the direction of causality. Second, the sample consisted of Hungarian couples and was not representative, therefore the results might be culturally specific or more relevant to a certain demographic. Cross-cultural aspects and bidirectional effects may be addressed in future research. Third, because of the greater dropout of couples in shorter relationships, our results might be generalizable for long-term relationships only. Fourth, because of eligibility criteria for the sample, the results cannot be generalized to couples where both partners are unemployed, or at least one of the partners a previous history of psychiatric disorders. Fifth, the current sample size limited the possible complexity of the model, but the further assessment of the couple’s relationship (e.g.: relationship satisfaction, distinguishing between self-oriented and partner-oriented goals) might enrich the research question and expand on the dyadic aspects of goal coordination and attainment. Lastly, not only romantic couples can be conceptualized as one self-regulating unit, but any interdependent close relationships, including coworkers, friends and family members as well. Further studies could broaden the scope and examine the connection between goal coordination, goal attainment and life satisfaction in other types of close relationships.

## Conclusions

Our results shed light on the additional benefit of accounting for the relationship context of individual goal pursuit in understanding life satisfaction. It emphasizes the importance of efficiency in goal coordination, as the effort only might be insufficient for life satisfaction. If partners feel that their goals are supported by their spouses, they might temporarily feel better, but this will preserve as long-term life satisfaction only if their goal coordination efforts are fruitful: when they can genuinely flourish together. In praxis, it might be highly beneficial to examine not only whether the couple coordinates their resources surrounding each other’s goals, but also facilitate the efficiency of their efforts.

## Data Availability

The datasets analyzed during the current study are available from the corresponding author on reasonable request.

## Appendix 1 Sensitivity Analysis for the Final APIMeM

To reduce the complexity of the model, we conducted a sensitivity analysis where we used the sum scores instead of latent factors for SWLS’, as SWLS showed high internal consistency. The resulting model had a good fit to the data (χ^2^ (92) = 115.157, CFI = 0.987 TLI = 0.983, RMSEA = 0.041, SRMSR = 0.068).

As the upper panel of Table

**Table 4:** Unstandardized effects for the simplified APIMeM with Project coordination as the independent variable, project attainment as mediator, and the aggregated scores for SWLS as outcome

	*b*	*SE*	*p*	CI (95%)	
				Upper	Lower
Direct effects					
Project coordination T1—Project attainment T2					
Actor	0.183	2.278	0.023	0.026	0.34
Partner	-0.041	-0.613	0.54	-0.17	0.089
Project attainment T2—SWLS T2					
Actor	0.27	5.428	< .001	0.172	0.367
Partner	0.242	4.505	< .001	0.137	0.347
Project coordination T1—SWLS T2					
Actor	-0.141	-1.909	0.056	-0.285	0.004
Partner	-0.012	-0.153	0.879	-0.162	0.139
SWLS T1—SWLS T2					
Actor	0.402	7.602	< .001	0.298	0.505
Partner	0.141	2.823	0.005	0.043	0.239
Indirect effects					
Actor					
Total	-0.101	-1.341	0.18	-0.249	0.047
Total Indirect	0.04	1.21	0.226	-0.025	0.104
Actor-Actor	0.049	2.033	0.042	0.002	0.097
Partner-Partner	-0.01	-0.592	0.554	-0.042	0.023
Partner					
Total	0.022	0.273	0.785	-0.133	0.177
Total Indirect	0.033	1.051	0.293	-0.029	0.095
Actor–Partner	0.044	2.077	0.038	0.002	0.086
Partner-Actor	-0.011	-0.601	0.548	-0.047	0.025

Unstandardized coefficients are reported

*SWLS* Satisfaction With Life Scale

*T1* First timepoint, *T2* second timepoint

Indirect effects were tested with bootstrap confidence intervals

4 shows, the simplified model has identical results to the final model. Higher project coordination resulted still in higher project attainment (b = 0.183, p = 0.023), which in turn was associated with higher life satisfaction (b = 0. 270, *p* < 0.001). The direct path between project coordination and life satisfaction was not significant (b = -0.141, p = 0.056). Compared to the final model, the partner effects are unchanged as well, as only the partner’s project attainment was associated with higher life satisfaction (b = 0.242, *p* < 0.001)- The path between the partner’s project coordination and project attainment was not significant (b = -0.041, p = 0.540) neither was significant the path between the partner’s project coordination and life satisfaction (b = -0.012, p = 0.879). All effects for life satisfaction between the first and the second timepoint were significant (b = 0.402, *p* < 0.001 for actor and b = 0.0.141, p = 0.005 for partner effects).

The lower panel of Table 4 presents the results from testing the indirect effects for the sensitivity analysis. The two patterns of complete mediations are still supported. Both the actor–actor (*b* = 0.049, p = 0.042). and the actor-partner indirect effects (*b* = 0.044, p = 0.038). were significant. All other indirect effects remained nonsignificant.
